# Suicide risk remission in collaborative care: a large-scale observational study

**DOI:** 10.3389/fpsyt.2025.1584753

**Published:** 2025-07-21

**Authors:** Carol Hardy, Virna Little, Brandn Green

**Affiliations:** ^1^ JG Research & Evaluation, Bozeman, MT, United States; ^2^ Concert Health, San Diego, CA, United States

**Keywords:** suicide prevention, collaborative care, suicide care pathway, suicide remission, suicide treatment, depression, risk assessment

## Abstract

**Introduction:**

Primary care settings represent a critical opportunity for suicide prevention, as many individuals who die by suicide visit primary care providers in their final month of life. The Collaborative Care Model (CoCM) offers a structured approach to behavioral health treatment in primary care, but research on its effectiveness for suicide risk management is limited. This study examined how clinical variables (days enrolled, clinical touchpoints, psychiatric consultations) relate to suicide risk outcomes in CoCM, and how these relationships are moderated by initial depression severity.

**Methods:**

Data from 3,599 patients with suicide risk flags who completed CoCM treatment were analyzed using ordinal logistic regression. Changes in suicide risk level from intake to discharge were categorized as improved, unchanged, or regressed.

**Results:**

Longer enrollment (OR=1.432, p<.001) and more clinical touchpoints (OR=2.584, p<.001) predicted improved outcomes. Higher baseline depression scores predicted poorer outcomes (OR=0.741, p<.001) but showed significant interaction with days enrolled. More psychiatric consultations (OR=0.813, p<.001) were associated with risk regression, likely reflecting appropriate escalation of complex cases.

**Discussion:**

CoCM shows promise for suicide risk management in primary care, with sustained engagement and frequent clinical contact improving outcomes. Results suggest treatment intensity should be tailored to initial depression severity.

## 1 Introduction

In the United States, suicide continues to be a primary cause of mortality for people between the ages of 10 and 64. The Centers for Disease Control and Prevention (1) reported that suicide ranked within the top nine causes of death in 2022. A dramatic upward trend in suicide rates occurred between 2000 and 2022, showing a 36% increase, with 2022 recording the highest number of suicide-related fatalities to date (1).

Research consistently demonstrates that many individuals who die by suicide did not access behavioral health services before their death (2, 3). A meaningful number of individuals who died by suicide had visited their primary care provider during their final month of life (4, 5). Additionally, patients who had previously disconnected from healthcare services often reconnect with primary care in their last month (6). These patterns suggest that primary care settings offer a crucial opportunity for identifying and supporting individuals at risk of suicide. The Collaborative Care Model (CoCM), which integrates behavioral health services directly into primary care settings through dedicated care managers and psychiatric consultants, provides a structured approach to capitalize on these critical intervention opportunities (7).

Suicide risk emerges from a complex interplay of clinical and social factors. Depression and anxiety disorders significantly increase suicide risk (8–10), while social determinants of health such as economic instability, lack of social support, and legal problems compound these clinical vulnerabilities (11, 12). Recent guidelines from the U.S. Preventive Services Task Force found insufficient evidence to recommend routine suicide risk screening in primary care, despite recommending screening for depression and anxiety (13, 14). This gap highlights the critical need for research examining suicide risk assessment and management in primary care populations, particularly studies that can inform evidence-based screening protocols and subsequent care pathways. This research need is especially pressing given that primary care remains a key point of contact for individuals at risk of suicide.

Meta-analysis has demonstrated CoCM’s effectiveness in managing depression outcomes by improving treatment adherence, symptom reduction and patient satisfaction (15–17), while systematic reviews show promising results for anxiety disorders (18). The model’s success extends across diverse populations and delivery methods, including virtual care settings (19). Randomized controlled trials have demonstrated CoCM’s effectiveness in treating depression across diverse populations and settings, including patients in the UK and Italy, as well as those with comorbid conditions such as diabetes and cardiovascular disease (15, 20–23). This broad evidence base for treating conditions strongly associated with suicide risk makes CoCM a promising framework for suicide prevention in primary care settings.

Research on CoCM’s impact on suicide risk is more limited, one randomized controlled trial demonstrated that older adults receiving CoCM showed greater reductions in suicidal ideation compared to those receiving usual care (24). Furthermore, the relationship between treatment “dose” and suicide-related outcomes in CoCM remains largely unexplored. This paper aims to add to the knowledge base by studying clinical variables that contribute to improved suicide treatment in a non-clinical CoCM setting.

Goal eleven of the Department of Health and Human Services’ Strategy for Suicide Prevention (25) is to “support research on suicide prevention.” Consistent with this goal, the researchers looked to understand whether there was a relationship between the “dose” of clinical touchpoints, psychiatric consults, and days in treatment with suicide risk acuity in Collaborative Care.

## 2 Materials and methods

### 2.1 Collaborative care

Collaborative care (CoCM) is an evidence-based model that identifies and treats patients with behavioral health conditions in healthcare settings. The Collaborative Care model aims to reduce barriers for patients requiring behavioral health treatment by caring for them alongside their trusted healthcare provider. The CoCM model incorporates several key components, including a patient registry for systematic tracking and monitoring, and a multidisciplinary team. This team consists of a healthcare provider, who serves as the initial point of contact and initially refers patients to Collaborative Care and may diagnose suicide risk; a psychiatric consultant, typically a master’s level clinician. For patients who are not improving or are worsening, the psychiatric consultant offers expert guidance to the care manager and healthcare provider. In the following analysis, this interaction is referred to as a psychiatric consultation. The psychiatric consultant can make pharmacological recommendations to the care manager. As a result, individuals identified with a behavioral health condition, including suicide risk, are referred to Collaborative Care by the healthcare provider. Some patients are identified at risk during a healthcare visit and others are identified during their Collaborative Care episode.

Patients enrolled in CoCM select from multiple treatment options and frequently choose more than one approach. Treatment options include both non-pharmacological interventions (behavioral activation, brief check-ins, talk treatment, symptom monitoring, goal setting, mindfulness strategies, symptom psychoeducation, and cognitive behavioral therapy) and medication-related support (medication adherence and medications support).

In 2017, Centers for Medicare and Medicaid Services (CMS) created dedicated current procedural terminology (CPT) codes for Collaborative Care which employs a monthly case rate payment structure that aligns with cumulative patient care activities throughout a calendar month. This patient-centered reimbursement approach facilitates high-frequency patient interactions, establishing CoCM as both a high touch and patient centered model. Intensive contacts may be particularly crucial for patients identified at suicide risk. The flexibility inherent in this reimbursement structure supports the comprehensive and responsive nature of the collaborative care model.

### 2.2 The suicide care pathway

Concert Health’s suicide safer care pathway guides treatment for patients identified as at-risk for suicide through measurement-based tools (PHQ-9 question 9, ASQ, C-SSRS) or clinician judgment. Once flagged as at-risk, patients remain flagged indefinitely due to evidence of sustained elevated risk for suicidal behaviors (26). The standard of care for flagged patients includes completion of the Stanley-Brown Safety Plan and lethal means restriction, tools designed to help patients manage crisis escalation (27).

**Figure 1:**
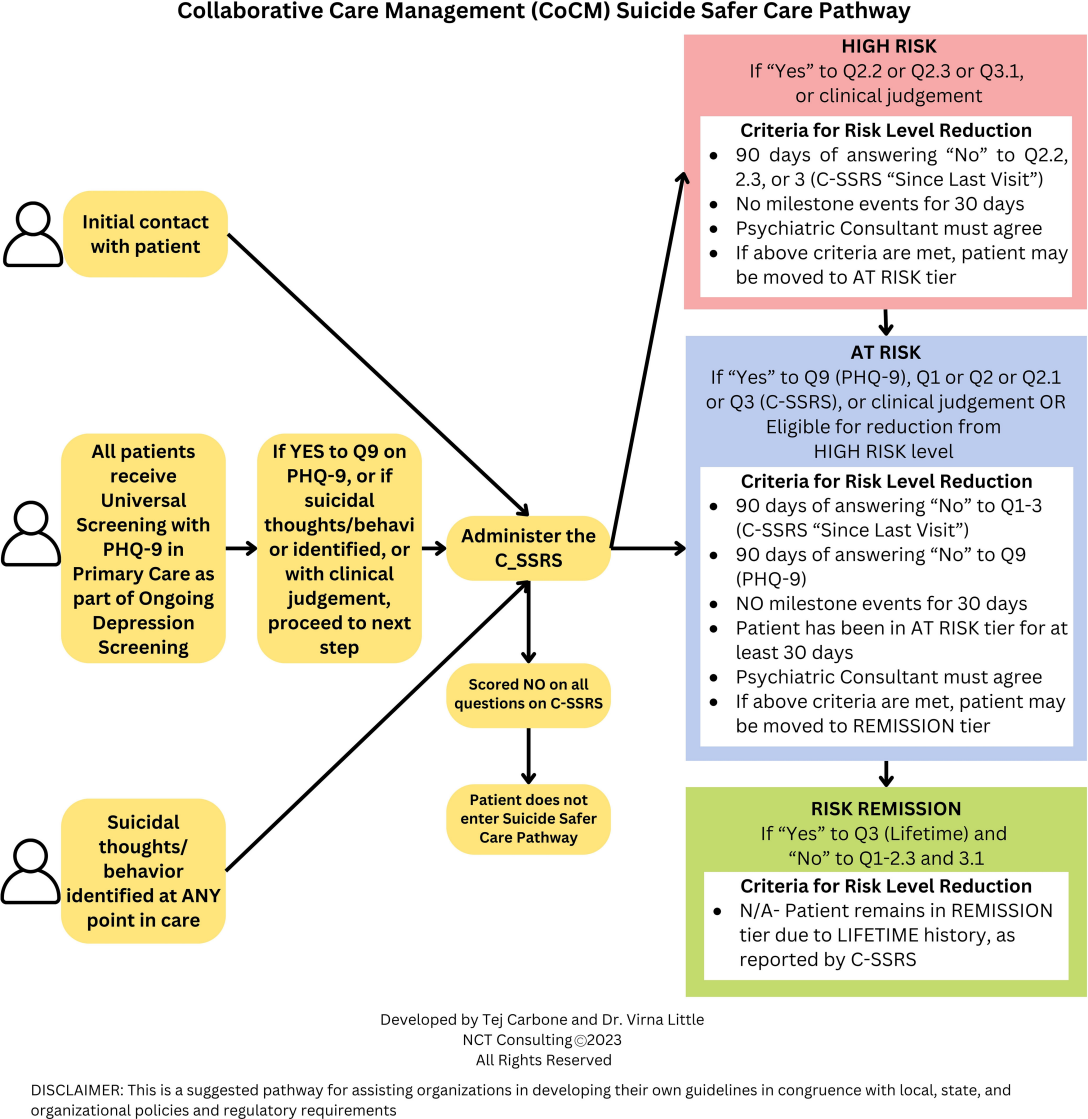
Collaborative care management (CoCM) suicide safer care pathway. **Figure 1** shows the Suicide Safer Care Pathways the Concert Health uses to standardize treatment for patients with suicide risk. For a patient to move from high risk to at risk, they must: have 90 or more days of answering no to 2.2, 2.3, or 3 on the C-SSRS; experience no milestone events (including but not limited to hospitalization, medication change, incarceration, geographic move, substance reuse, etc.); and receive agreement from the psychiatric consultant with the care manager’s assessment. For a patient to move from at risk to risk remission, they must answer no to questions 1 through 3 of the C-SSRS, answer no to question 9 of the PHQ-9, experience no milestone events for 30 days, and receive agreement from the psychiatric consultant with the care manager’s assessment.

The pathway stratifies patients into three tiers: High Risk, At Risk, and Risk Remission (Historical risk). Movement to lower risk tiers requires meeting specific criteria: negative C-SSRS responses for three consecutive months, stable personal circumstances, and care team consensus. Patients experiencing life transitions, medication changes, or active psychotic features are not eligible for risk reduction. See **Figure 1** for more details.

#### Outcome of interest

The primary outcome of interest in this analysis is whether a patient progressed to a lower tier before discharging from care.

#### Patient classification

Patients are classified into three categories based on their risk level changes:

##### Improved risk flag

Patients who moved down at least one suicide risk tier. Example: A patient who transitioned from High Risk to At Risk, or from At Risk to Risk Remission.

##### No change in risk flag

Patients who maintained their initial risk level throughout their care. Example: A patient who entered and exited the suicide care pathway as At Risk.

##### Risk flag regressed

A small percentage of patients who discharged from care with an elevated risk level compared to their entry level. Example: A patient who entered the suicide care pathway as At Risk but discharged as High Risk.

Concert Health is a behavioral health organization that provides CoCM services to primary care and other medical providers in 17 states. This study included 52 healthcare organizations, including hospital systems, medical groups, community health centers, and independent practices, all providing CoCM. The results of this study are generalizable to patients at risk for suicide receiving treatment within Collaborative Care Model settings. The Suicide Care Pathway used closely resembles the widely implemented Zero Suicide (28) framework, enhancing the applicability of our findings to similar suicide prevention approaches in primary care.

Between November 24, 2021, and December 31, 2023, 3,599 patients treated with the Suicide Risk Pathway within the Concert Health electronic health system were analyzed. Patients were included in the study if they were not missing variables of interest and had a closed treatment period. The study outcome was the aforementioned patient risk classification. The clinical variables of interest were length of treatment, number of clinical touchpoints, and number of psychiatric consultations. Length of treatment is defined as the number of days a patient was enrolled before their treatment period ended. A clinical touchpoint is an encounter with a behavioral care manager lasting 5 or more minutes, with the vast majority conducted virtually, though face-to-face were also considered. Follow-up via messaging is not considered a clinical touchpoint and was not included in the analysis. A psychiatric consultation occurs when a psychiatric expert provides guidance to the behavioral care manager and healthcare provider. The researchers controlled for potential confounders including PHQ-9 baseline (a proxy for initial patient severity), insurance type (with Medicaid serving as a proxy for income), age group, and diagnosis category (Depression or other – including anxiety). Age was categorized into discrete groups to allow for interpretation of effects across broader age ranges rather than as a continuous variable. Demographics such as race, gender, and ethnicity were not available at the time of the study because these data were not being collected. The absence of these demographic variables represents a potential source of bias that reduces the findings generalizability.

### 2.3 Analysis plan

Analyses were conducted in R using various R packages for data cleaning, analysis, and presentation (29–34). An ordinal logistic regression was used to test whether there were associations between clinical variables of interest and suicide risk improvement. Specifically, understanding whether there was relationship between the “dose” of clinical touchpoints, psychiatric consults, and days in treatment with suicide risk acuity at discharge. Continuous variables were centered and standardized, making coefficient interpretations relative to the sample mean rather than zero. For example, the effect of days enrolled is interpreted in relation to the average enrollment duration rather than zero days enrolled to enhance interpretability. The ordinal logistic regression model is an extension of the binary logistic regression, designed to accommodate dependent variables with ordered categorical outcomes (35). In this study, the dependent variable of interest is the change in patient suicide risk, categorized into three ordered levels: regression (deterioration), stability (no change), and improvement. This model preserves the ordinal nature of these outcomes while making fewer assumptions than linear regression. Unlike multinomial logistic regression, which would treat the three outcomes as unordered categories, ordinal regression leverages their inherent ordering. The proportional odds assumption was tested and found reasonable for our data.

The researchers aimed to isolate the associations between patient suicide risk and three clinical variables: days enrolled, psychiatric consultations received, and clinical touchpoints received. Additionally, the researchers sought to understand how these effects might be moderated by the severity of the initial depression assessment. To examine these complex relationships, two models were fit: one with main effects only and another that included interactions between baseline PHQ-9 scores and the clinical variables. The researchers controlled for potential confounders including insurance type, age group, and diagnosis category (depression or other – including anxiety). A sensitivity analysis was conducted to assess the possibility of influential outliers.

The sample size was determined by the number of eligible patients in the electronic health record database rather than *a priori* power calculations. A complete case analysis approach was used, meaning patients with missing values for any variables of interest were excluded from the study. This approach ensured that all analyses were conducted on a complete dataset. Data imputation methods were not employed because the pattern of missingness was not random, which could have introduced bias if imputed.

An IRB was submitted to Western IRB and determined to be exempt under 45 CFR § 46.104(d) (4).

## 3 Results

### 3.1 Participants

**Figure 2:**
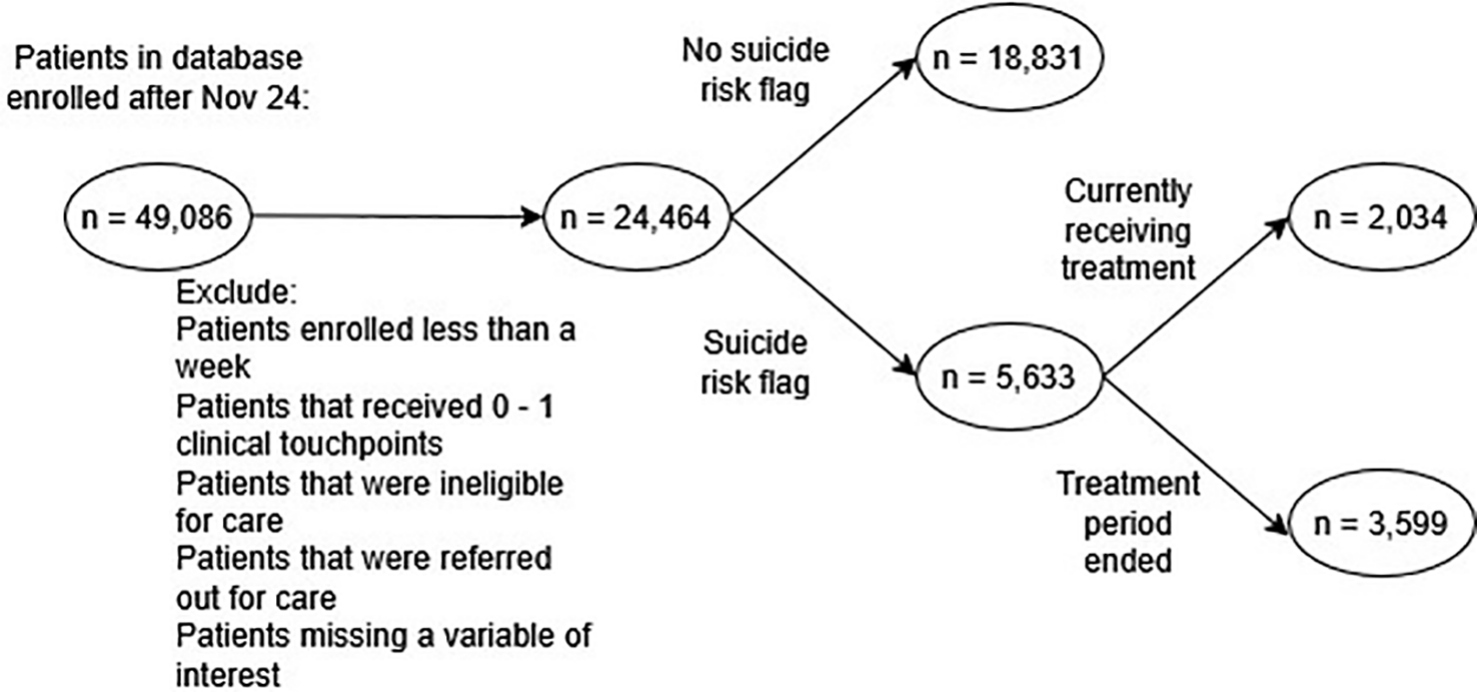
Sample diagram. **Figure 2** shows the flow of patients included in the analysis. The Concert Health electronic health system database had 49,086 patients records whose enrollment began after November 24, 2021. These patients were filtered through the initial screening criteria. Patients were removed if they did not actually receive care within the system (too few days enrolled, too few touchpoints, ineligible for care or referred out for care). If a patient was missing any variable of interest, they were removed during initial selection. Finally, only patients flagged at risk for suicide with closed treatment periods were included, resulting in a final sample size of 3,599.

Between November 24, 2021 and December 31, 2023, 49,086 patients were enrolled in the Concert electronic health record database. Of these, 24,464 met the initial inclusion criteria. 5,633 of the patients had a suicide risk flag. 3,599 of the patients had a closed treatment period. **Figure 2** shows the patient inclusion diagram.

• Exclusion criteria:

- If a variable of interest was unknown/unrecorded
- Patients enrolled for less than a week
- Patients that received 0–1 clinical touchpoints
- Patients that were ineligible for care
- Patients that were referred out for care

### 3.2 Demographics

**Figure 3:**
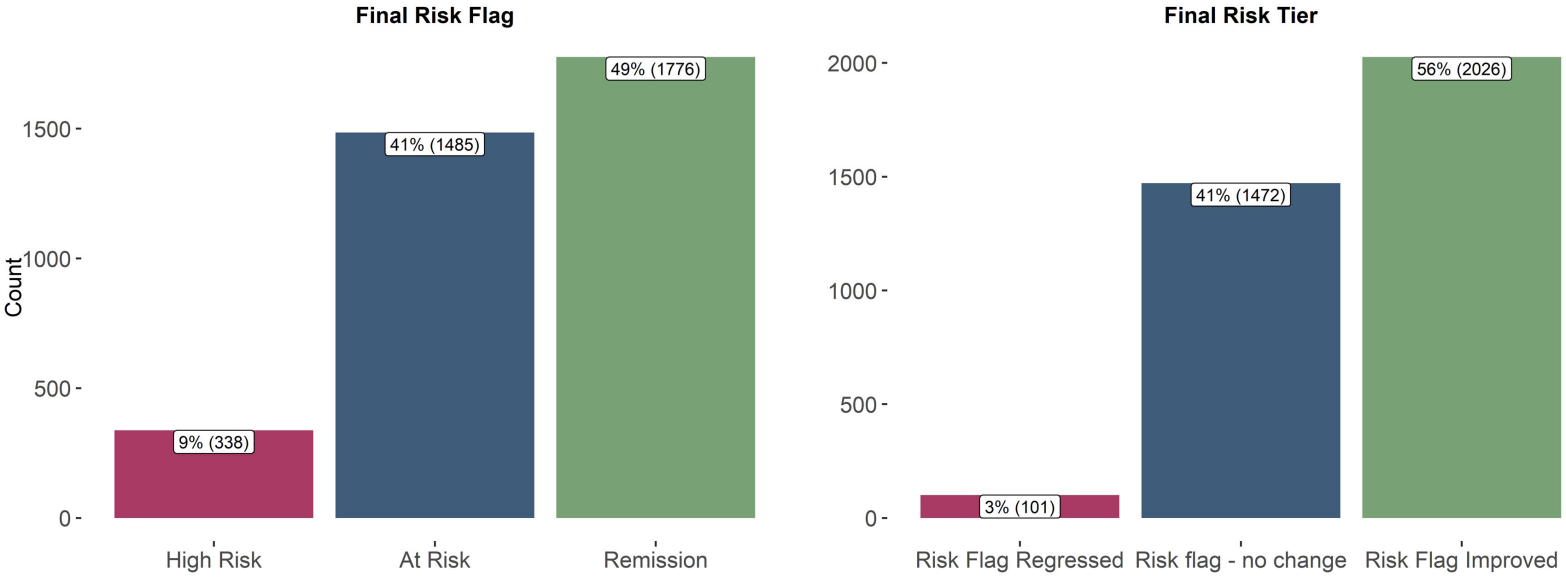
Patients’ Final Risk Flag and Final Risk Level (n = 3,599). **Figure 3** shows the risk flag and tier of discharged patients in the study. A patient’s final risk flag can fall into three categories: high risk (most acute), at risk (present risk), or remission (no currently detected risk). Based on changes between initial and final risk levels, patients were classified as having an improved risk flag (moved down at least one tier), no change in risk flag (maintained initial level), or regressed risk flag (discharged with an elevated risk compared to entry level).

**Figure 3** summarizes the risk level of patients discharging from care. Approximately half of the sample (n = 1,776) achieved the primary goal of the pathway, being discharged from care with a risk remission flag, indicating a substantial reduction in suicide risk. A majority of the participants (n = 2,026) demonstrated improvement in their risk flag categorization throughout the course of care.

The researchers aim to investigate the relationship between specific clinical variables and patients’ final risk tier, with a particular focus on identifying which variables, and at what levels, mediate this outcome. **Table 1** summarizes the descriptive statistics for the total sample and stratified by final risk tier groups. For continuous variables, the mean, median, and standard deviation are presented. For categorical variables, the distribution is presented as percentages across categories. The observed discrepancies between mean and median values suggest the presence of outliers in the sample, specifically patients who received substantially more care than average.

**Table 1:** Demographics of patients used in the analysis (n = 3,599).

Demographic characteristic	Statistic	Total	Risk flag regressed	Risk flag - no change	Risk flag improved
Days Enrolled	Mean	164	178	112	200
	Median	124	118	86	165
	SD	127	149	88	136
	IQR	138	176	80	158
Number of Psychiatric Consultations	Mean	3	6	2	4
	Median	2	5	1	3
	SD	4	6	3	4
Clinical Touchpoints	IQR	3	5	2	4
Clinical Touchpoints	Mean	1	1	6	14
PHQ-9 Baseline	Mean	16	18	16	15
	Median	16	18	17	15
	SD	6	5	5	6
Insurance Type	Commercial	1,907 (52.99%)	46 (45.54%)	746 (50.68%)	1,115 (55.03%)
	Medicaid	1,112 (30.9%)	39 (38.61%)	503 (34.17%)	570 (28.13%)
	Medicare	580 (16.12%)	16 (15.84%)	223 (15.15%)	341 (16.83%)
	Total	3,599 (100.01%)	101 (99.99%)	1,472 (100%)	2,026 (99.99%)
Age Group	17 and under	264 (7.34%)	5 (4.95%)	99 (6.73%)	160 (7.9%)
	18–30 years	1,190 (33.06%)	45 (44.55%)	495 (33.63%)	650 (32.08%)
	31–45 years	952 (26.45%)	17 (16.83%)	405 (27.51%)	530 (26.16%)
	46–64 years	820 (22.78%)	24 (23.76%)	341 (23.17%)	455 (22.46%)
	65+ years	373 (10.36%)	10 (9.9%)	132 (8.97%)	231 (11.4%)
	Total	3,599 (99.99%)	101 (99.99%)	1,472 (100.01%)	2,026 (100%)
Diagnosis Category	Anxiety Disorder	977 (27.15%)	19 (18.81%)	407 (27.65%)	551 (27.2%)
	Depressive Disorder	2,480 (68.91%)	77 (76.24%)	1,006 (68.34%)	1,397 (68.95%)
	Other	142 (3.95%)	5 (4.95%)	59 (4.01%)	78 (3.85%)
	Total	3,599 (100.01%)	101 (100%)	1,472 (100%)	2,026 (100%)

**Table 1** summarizes the descriptive statistics for the total sample and stratified by final risk tier groups. For continuous variables, the mean, median, and standard deviation are presented. For categorical variables, the distribution is presented as percentages across categories. Totals may not equal 100% because of rounding errors.

### 3.3 Clinical variables and suicide risk plots

**Figure 4:**
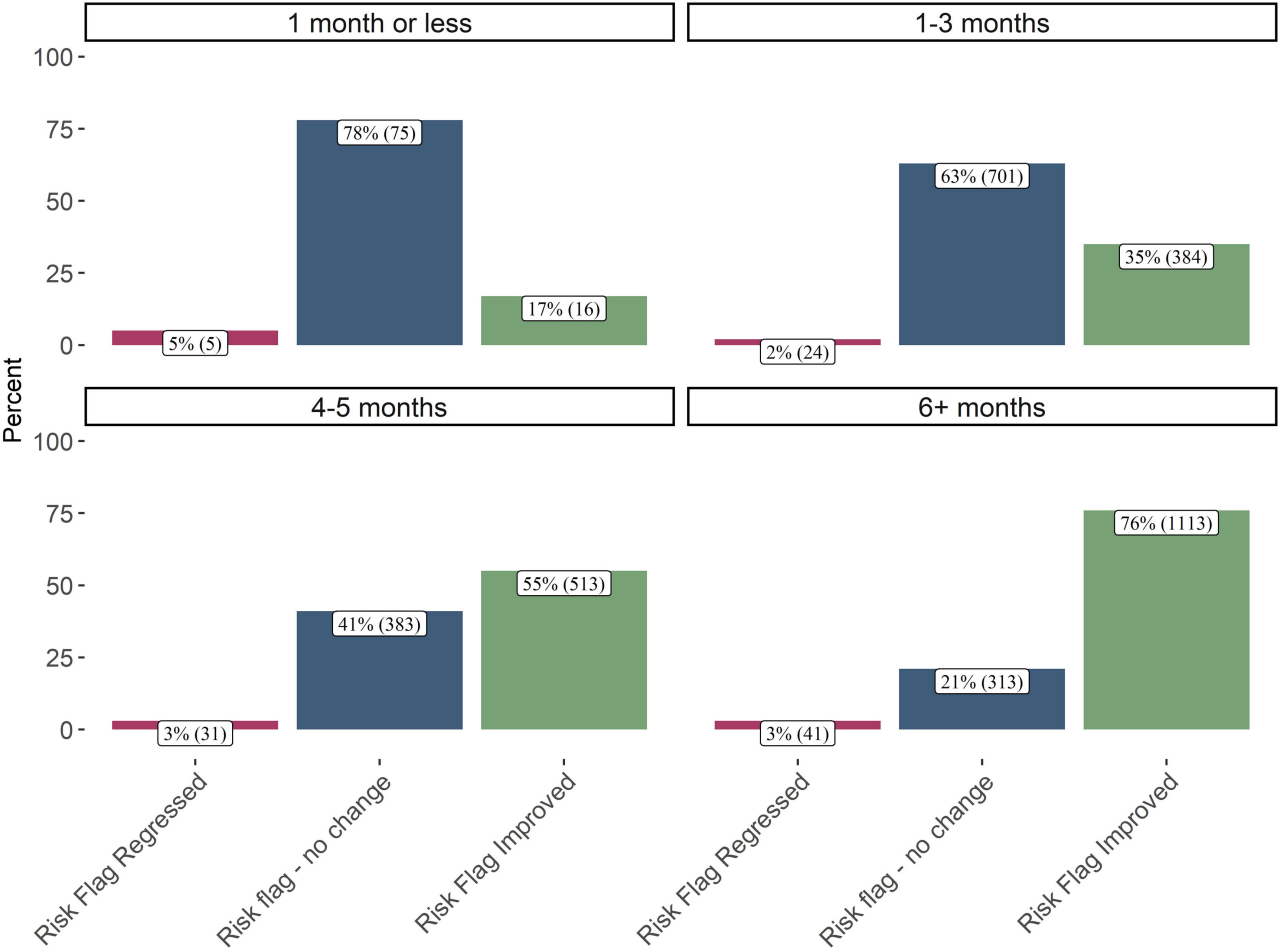
Final risk flag by days enrolled in collaborative care (n = 3,599). This plot shows patients’ discharge status compared to the time spent enrolled in CoCM. 76% of patients that spent 6 or months in CoCM discharged with a risk flag improvement. In contrast 78% of patients that were enrolled in CoCM for 1 month or less discharged with no change in risk flag status. This plot suggests that patients enrolled in CoCM for longer durations were much more likely to see improved suicide risk.

**Figure 5:**
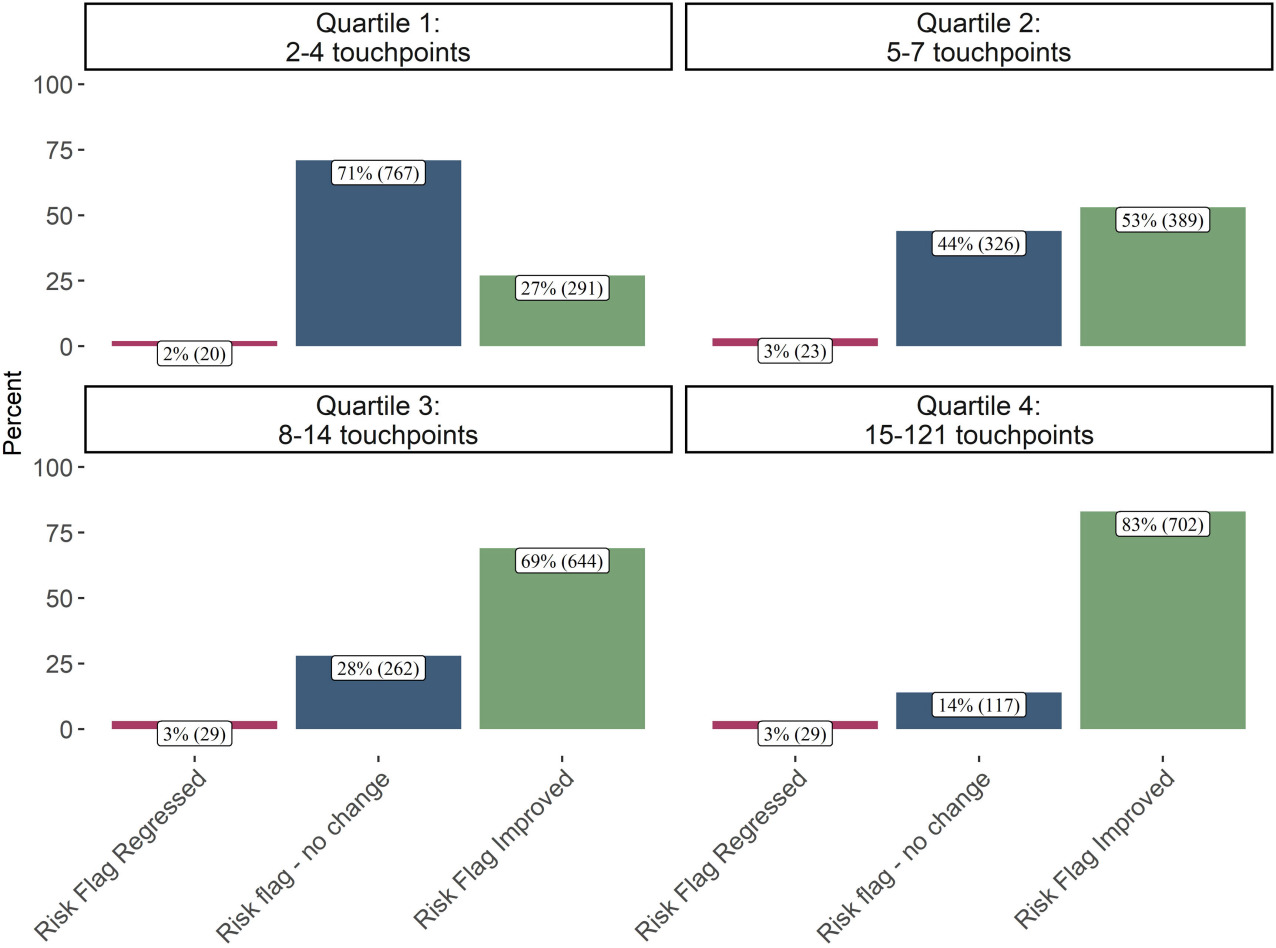
Final risk flag by clinical touchpoints received (n = 3,599). This plot shows patients’ discharge status compared to clinical touchpoints received. 83% of patients that received the most clinical touchpoints discharged with an improved risk flag. In contrast 71% of patients that received the fewest clinical touchpoints were discharged with no change in risk flag. This plot suggests the patients’ receiving the most clinical touchpoints are also the most likely to see improved suicide risk.

**Figure 6:**
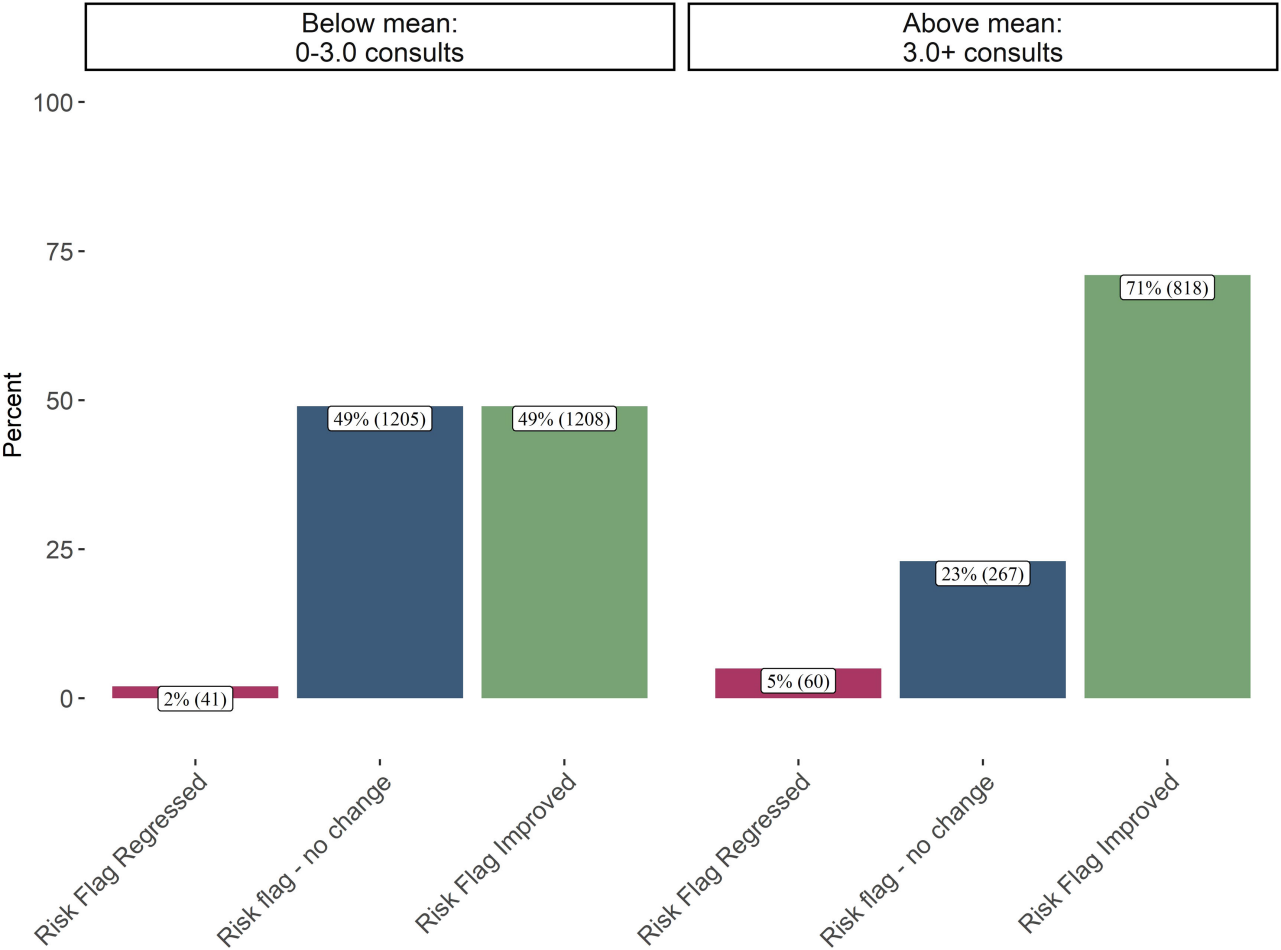
Final risk flag by psychiatric consultations received (n = 3,599). This plot shows patients’ discharge status compared to psychiatric consultations. 71% of patients that received the most psychiatric consultations discharged with an improved risk flag. In contrast 49% of patients that received the fewest psychiatric consultations were discharged with no change in risk flag. This plot suggests that patients’ receiving an above average number of psychiatric consultations are less likely to see no change in their risk flag.

As part of the investigation, researchers examined the potential associations between suicide risk improvement and three key variables: duration of enrollment, frequency of clinical touchpoints, and number of psychiatric consultations. To visualize these relationships, the following plots (**Figures 4**–**6**) illustrate the percentage of patients who demonstrated an improvement in their suicide risk scores, stratified by either length of time in treatment, number of clinical touchpoints received, or number of psychiatric consultations received.

### 3.4 Model results

**Tables 2**, **3** show the odds ratios from the main effects and interaction ordinal logistic regressions.

**Table 2:** Odds ratios from main effects ordinal logistic regression (n = 3,599).

Variable Description	Variable	Odds Ratio	Confidence Interval
Clinical Variables	Days Enrolled	1.432***	(1.2119, 1.6958)
	PHQ-9 Baseline Score	0.7409***	(0.6877, 0.7977)
	Number of Psychiatric Consultations	0.8126***	(0.722, 0.9152)
	Clinical Touchpoints	2.5845***	(2.1171, 3.169)
Age Groups	Age 18–30 years	0.9384	(0.6994, 1.2555)
	Age 31–45 years	1.0774	(0.7973, 1.4524)
	Age 46–64 years	1.0021	(0.734, 1.365)
	Age 65+ years	1.2942	(0.8448, 1.9828)
Insurance	Medicaid Insurance	0.7458***	(0.6355, 0.875)
	Medicare Insurance	0.8137	(0.616, 1.0753)
Diagnosis	Depressive Disorder Diagnosis	1.0458	(0.8882, 1.2311)
	Other Diagnosis	0.9128	(0.6222, 1.3431)

Asterisks denote levels of statistical significance: ***p < 0.001, **p < 0.01, *p < 0.05, with unmarked coefficients indicating p ≥ 0.05. The odds ratio indicates the odds of the patients’ suicide risk flag staying the same or improving versus regressing. Values above 1.0 indicating increased odds of improvement and values below 1.0 indicating decreased odds of improvement.

**Table 3:** Odds ratios from interaction ordinal logistic regression (n = 3,599).

Variable description	Variable	Odds Ratio	Confidence Interval
Clinical Variables	Days Enrolled	1.375***	(1.1597, 1.6337)
	PHQ-9 Baseline Score	0.7309***	(0.6717, 0.7944)
	Number of Psychiatric Consultations	0.8081***	(0.7142, 0.9161)
	Clinical Touchpoints	2.7493***	(2.2301, 3.4059)
Age Groups	Age 18–30 years	0.9344	(0.6961, 1.2507)
	Age 31–45 years	1.068	(0.79, 1.4404)
	Age 46–64 years	0.9917	(0.726, 1.3516)
	Age 65+ years	1.2711	(0.8289, 1.9492)
Insurance	Medicaid Insurance	0.7443***	(0.6341, 0.8735)
	Medicare Insurance	0.8155	(0.6173, 1.078)
Diagnosis	Depressive Disorder Diagnosis	1.0473	(0.8892, 1.2332)
	Other Diagnosis	0.9077	(0.6186, 1.3356)
Interactions	Days Enrolled x PHQ-9 Baseline	1.2329*	(1.0315, 1.4723)
	Number of Psychiatric Consultations x PHQ-9 Baseline	0.9952	(0.8778, 1.1254)
	Number of Clinical Touchpoints x PHQ-9 Baseline	0.7825*	(0.6325, 0.9672)

Asterisks denote levels of statistical significance: ***p < 0.001, **p < 0.01, *p < 0.05, with unmarked coefficients indicating p ≥ 0.05. The odds ratio indicates the odds of the patients’ suicide risk flag staying the same or improving versus regressing. Values above 1.0 indicating increased odds of improvement and values below 1.0 indicating decreased odds of improvement.

#### 3.4.1 Main effect model coefficient interpretations

- For patients that receive ~294 days of care, (one standard deviation [127 days] above the mean [167 days]), the odds of their risk flag improving or staying the same (versus a regression) increases 43% (multiplied 1.43 times), holding all other variables constant (p < 0.001).
- For patients that receive ~6 psychiatric consultations, (one standard deviation [3] above the mean [3]), the odds of their risk flag staying the same or improving (versus a regression) is 19% (1-0.812) lower, holding all other variables constant (p < 0.001).
- For patients that receive ~22 clinical touchpoints (one standard deviation [11] above the mean [11]), the odds of their risk flag staying the same or improving (versus a regression) is multiplied by 2.58 times (158% increase) (p < 0.001), holding all other variables constant.
- For patients whose baseline PHQ-9 is ~22 (one standard deviation [6] above the mean [16]), the odds of their risk flag staying the same or improving (versus a regression) is 26% (1-0.74) lower (p < 0.001), holding all other variables constant.
- For patients on Medicaid, as compared to commercial or Medicare, the odds of their suicide risk staying the same or improving is 25% (1-.75) lower (p < 0.001), holding all other variables constant.

**Figure 4** shows that 76% of patients enrolled for 6 or more months improved their suicide risk score. The ordinal logistic regression confirmed the positive association between days enrolled and a stable or improved risk flag even after controlling for clinical touchpoints, PHQ-9 baseline, days enrolled, age, insurance, diagnosis, and after accounting for potential outliers. The interaction regression analysis also uncovered an interaction effect: patients who entered treatment with higher (more severe) initial PHQ-9 scores appeared to derive greater benefit from more days enrolled. This finding suggests that longer treatment periods may be particularly advantageous for those presenting with more severe depressive symptoms at the outset of care.

**Table 2** shows patients whose risk flag improved had on average more clinical touchpoints. **Figure 5** also showed that 83% of patients receiving the highest quartile of touchpoints improved their suicide risk flag. The ordinal logistic regression also confirms this association with a very large effect size demonstrating that after controlling for all the variables in the model, more clinical touchpoints are associated with 158% increase in the odds of the risk flag staying the same or improving. An interaction was tested between patients with a higher PHQ-9 baseline and more clinical touchpoints. This interaction test revealed, with statistical significance, that more clinical touchpoints may be more beneficial for patients with a lower initial baseline PHQ-9.

Patients entering care with a more severe PHQ-9 are less likely to see no change or improve their suicide risk score compared to patients that enter care with less severe acuity, after controlling for all the other variables in the model. Patients on Medicaid are also less likely see no change or an improvement when compared to commercial and Medicare patients after controlling for all the other variables. None of the coefficients for age or diagnosis category are statistically significant, indicating that CoCM may be equally effective for all age groups and anxiety, depression, and other diagnoses.

Psychiatric consultations are typically initiated when a patient is not improving as expected all allow for the care manager to seek guidance from an experienced clinician. **Table 1** shows that patients who regressed during care received a median of 5 psychiatric consultations, compared to 3 for those who improved. The ordinal logistic regression showed statistically significant association between patients that receive more psychiatric consults and terminating care with a regression, after accounting for number of clinical touchpoints, days enrolled, age, insurance etc. **Figure 6** illustrates this relationship, showing that 5% of patients receiving above-average consultations concluded care with regression, compared to only 2% among those with fewer consultations. The higher rate of consultations for patients who ultimately regress implies a proactive approach to addressing complex cases, though it also highlights the persistent challenges in treating more severely depressed or complex patients.

### 3.5 Analysis without influential points

Given differences between mean and median values for days enrolled and clinical touchpoints received, the researchers investigated the possibility of influential outliers in the ordinal logistic regression. Leverage values were calculated using the hat matrix from the model, with high leverage points identified as those exceeding twice the mean leverage value. The model estimates were then re-calculated excluding these high leverage observations (n = 299).

The sensitivity analysis excluding high leverage observations yielded similar patterns to the primary analysis, supporting the robustness of our main findings. While most coefficients remained stable, the association between clinical touchpoints and improved strengthened, with the odds ratio increasing from 2.75 to 3.29. The positive effect of extended enrollment duration also remained consistent, with a slight increase in the odds ratio from 1.38 to 1.46. The relationship between Medicare insurance status and outcomes showed the largest change, with the odds ratio decreasing from 0.82 to 0.73, suggesting this effect may be less stable. Overall, the exclusion of influential points did not substantially alter the core conclusions about the effectiveness of clinical touchpoints and sustained enrollment in improving suicide risk outcomes.

## 4 Discussion

Similar to previous findings (36–38) showing that increased clinical touchpoints improve depression and anxiety outcomes across various populations, we found that more frequent clinical contact was strongly associated with improved suicide risk status. The study also reinforces earlier findings regarding insurance-based disparities, with Medicaid patients showing lower odds of risk improvement compared to those with commercial insurance (37, 38). The disparate outcomes observed among Medicaid recipients likely stem from multiple interconnected factors. Medicaid recipients report barriers in access to care and tend to be more likely to experience mental illness and poorer baseline health (39–41) compared to non-Medicaid recipients. Additionally, despite half of Medicaid beneficiaries belonging to a racial and ethnic minority group, studies have shown that minority enrollees report having worse experiences (42–44).

While previous research found that longer enrollment could be associated with poorer outcomes for depression and anxiety (36), in the case of suicide risk management, sustained engagement appears beneficial. These findings suggest that suicide risk management may require a more intensive and sustained treatment approach compared to general depression and anxiety care in the collaborative care model.

Our finding that patients with higher baseline PHQ-9 scores were less likely to show improvement in suicide risk aligns with established literature on depression treatment outcomes. Both the STAR*D (45) trial and Katon’s (46) analysis of collaborative care demonstrated that patients with greater initial severity required more intensive treatment but were still less likely to achieve remission, despite receiving more clinical attention and resources. Our analysis showed that patients receiving more psychiatric consultations were less likely to improve their suicide risk. Patients requiring psychiatric consultation are more clinically complex or serious cases, thus our finding supports the STAR*D and Katon results. The interaction analyses revealed nuanced relationships between baseline severity and treatment elements. Patients with higher initial PHQ-9 scores appeared to benefit more from extended treatment duration, suggesting that longer enrollment may be particularly important for more severely depressed patients. Conversely, clinical touchpoints appeared more beneficial for patients with lower baseline PHQ-9 scores, indicating that treatment intensity may need to be tailored based on initial depression severity. However, more research is needed to confirm these findings.

Our findings have important implications for suicide prevention in primary care settings. While the U.S. Preventive Services Task Force currently finds insufficient evidence to recommend routine suicide risk screening (14), our results suggest that when screening is coupled with a structured care pathway and robust follow-up system like CoCM, meaningful risk reduction is possible. The high rate of risk improvement among patients receiving frequent clinical touchpoints demonstrates that CoCM’s systematic approach to monitoring and treatment can effectively support patients identified through screening. This may help address documented provider hesitancy to screen for suicide risk, as CoCM offers a clear pathway for managing identified patients, including regular risk assessment, standardized care protocols, and access to psychiatric consultation for complex cases.

Collaborative Care is an evidence-based model whose core components support effective and comprehensive suicide safer care. With research supporting that many individuals seek care and/or are identified in primary care affords an opportunity to connect individuals at risk to evidence based treatment alongside their trusted healthcare provider. For many, this could mean access to treatment, particularly for those in rural and underserved communities, where there is little access to behavioral health providers. Collaborative Care offers a unique opportunity to provide options for healthcare providers struggling with access to behavioral health treatment for their patients. Simultaneously many providers report hesitancy to screen for suicide risk due to lack of referral and treatment resources (47, 48), Collaborative Care offers potential relief for this struggle (49).

Several limitations should be considered when interpreting these findings. As an observational study examining real-world clinical data, we cannot make causal claims about CoCM’s effectiveness compared to other approaches. However, our findings are consistent with previous randomized controlled trials. The IMPACT trial (n = 1,801), which studied CoCM’s effect on suicide risk in older adults, found that patients receiving usual care showed no improvement in suicidal ideation over two years and even experienced slight increases (50). Similarly, the PROSPECT study (n = 599) found that patients receiving CoCM were more than twice as likely to experience reductions in suicidal ideation compared to usual care, with higher treatment engagement rates (85-89% versus 49-59%) for antidepressants and psychotherapy (24). Future research would benefit from the use of a comparison group to compare the impacts of CoCM to care-as-usual. Additional limitations include the absence of demographic variables such as race, ethnicity, and gender, preventing analysis of potential care disparities; the potential loss of nuanced changes in suicide risk due to our three-tier categorization system; and possible selection bias, as patients who remained in treatment longer may differ systematically from those who discontinued early or were referred out for care. Variables that we were unable to control for may have confounded the results of the study. Some examples include medication adherence, treatment choices, history of suicide attempts, and demographic characteristics mentioned above.

## 5 Conclusion

These findings have important clinical implications for suicide risk management in primary care settings. Extended treatment periods and frequent clinical touchpoints were associated with improved outcomes, particularly for patients with higher initial depression severity, supporting CoCM’s monthly payment model that enables regular patient contact. The disparate outcomes for Medicaid patients highlight the need for targeted support for this vulnerable population, while the pattern of psychiatric consultations reflects appropriate clinical judgment in escalating complex cases. Overall, these results demonstrate CoCM’s value as a structured framework for suicide prevention in primary care settings. Future research should examine the optimal (51)implementation of clinical touchpoints and psychiatric consultations, particularly during the initial months of treatment when patients may be at highest risk.

## Data Availability

The data analyzed in this study is subject to the following licenses/restrictions: The data are sensitive in nature (suicide risk patients). Data could be made available upon request with the approval of Concert Health. Requests to access these datasets should be directed to carol@jgresearch.org.
